# A yearly maximum sea level simulator and its applications: A Stockholm case study

**DOI:** 10.1007/s13280-021-01661-4

**Published:** 2021-11-20

**Authors:** Magnus Hieronymus

**Affiliations:** Swedish Meteorological and Hydrological Institute, Norrköping, Sweden

**Keywords:** Adaptation, Extreme sea levels, Flooding, Sea level rise, Stockholm

## Abstract

A yearly maximum sea level simulator for Stockholm is presented. The simulator combines extreme sea level estimates and mean sea level rise projections into a joint probabilistic framework. The framework can be used, for example, to assess the risk that new structures placed at the current minimum allowed height above the sea level can become flooded in the future. Such assessments can be used to underpin future building free levels, which would be a great improvement over the much more arbitrary criteria in use today. Another strong point of the framework is that it can be used to quantify the influence of uncertainties in mean sea level projections, estimates of sea level extremes and future emission scenarios on the risk of flooding. For Stockholm mean sea level uncertainty is found to be much more important than extreme sea level uncertainty. The framework is also set-up to test adaptation measures. It is found that protections that are built once the mean sea level has risen above some given threshold can be very efficient. Lastly, the framework is embedded into a simple decision problem that can be used to calculate risk/reward ratios for land development as a function of height above today’s mean sea level.

## Introduction

Infrastructure planning in many coastal areas around the world is complicated by rising mean sea levels (Hinkel et al. 2018; Oppenheimer et al. 2019). Rising mean sea levels increase the risk of coastal flooding, and the great inertia of the ice-sheets and the deep ocean assures that sea levels will continue to rise long after atmospheric greenhouse gas concentrations have stabilized (Fox-Kemper et al. 2021). Some key difficulties that must be tackled in coastal infrastructure plans, apart from the long time scales, are that: sea level rise is inhomogeneous in space, future sea level projections are highly uncertain and sea level projections depend on poorly constrained future emission, concentration or temperature pathways (van Vuuren et al. 2011; Mitrovica et al. 2018; Oppenheimer et al. 2019). Apart from mean sea level rise; successful infrastructure planning must also handle extreme sea levels that can occur, for example, during storms when the sea level temporarily can become elevated far above its mean level. Plans must therefore be made with regional or even local scales in mind. Planning for sea level rise with local scales in mind is, however, challenging and can be costly. Many, and especially smaller, municipalities also have limited expertise and lack adequate tools and data to make such plans (Schöld et al. 2020).

The focus of this manuscript is to introduce a new tool, a yearly maximum sea level simulator, aimed to aid such planning. The simulator is set-up for Stockholm, which is used as a case study, but the same methodology can be used anywhere. Stockholm similarly to most Swedish coastal cities expects a sea level rise that is considerably smaller than the global average. The comparably small sea level rise owes primarily to a significant post glacial rebound, but also to Sweden’s relative proximity to Greenland, which ensures that melting of the Greenland ice sheet has a small influence on Swedish sea levels (Hieronymus and Kalén 2020). Mean sea level rise projections and extreme sea level estimates were recently produced for a number of Swedish locations including Stockholm by Hieronymus and Kalén (2020), and those projections and estimates will be used here. The focus here is, however, not on the specifics of these data, but rather on how to combine them into a framework that can be used to produce probabilistic answers to a number of important sea level related questions. Such as, for example, what land is suitable for new settlements and how effective certain adaptation measures might be.

Current practise in Stockholm is that new developments should be placed on land laying higher than 2.7 m in the national height system RH2000 (Department of civil engineering county administrative board of Stockholm 2015). This is approximately equal to 2.5 m above the current mean sea level in Stockholm, since the 1986-2005 averaged sea level in Stockholm was 19 cm in RH2000 (Swedish Meterological and Hydrological Institute 2021). The rational for choosing this particular level is not given in the reference, which is only available in Swedish. However, it is said to be composed of an assumed 1 m sea level rise until the year 2100, the estimated local land rise during the same period, a 100 year return level and an extra safety margin of 0.9 m. Uncertainty is not treated specifically and neither is it specifically stated what level of risk the 2.7 m building free level level is supposed to protect against. Swedish municipalities generally speaking enjoy greater freedom in choosing their own building free levels than municipalities do in many other countries. The recipe of adding some return level to some assumed mean sea level rise and sometimes adding an extra safety margin is, however, commonly used in many countries (Arns et al. 2017; Schöld et al. 2020)

The simulator framework proposed here, in contrast to the current practise, is well tailored to handle uncertainties and it can easily be used, for example, to calculate the risk that land situated 2.5 m above the current mean sea level will be flooded sometime between now and the year 2100. The results are, however, contingent on our beliefs in the likelihood of different climate scenarios coming to pass. The simulator can also give quantitative answers to questions such as how does the assumed likelihood of a given climate scenario coming to pass affect the probability that a given level will be flooded. Another application is to quantify the risk that a certain level becomes flooded given that protection is put in place when the mean sea level has risen by some set amount.

The main aim of the manuscript is to introduce the new simulator framework that combines mean level rise and sea level extremes into one joint probabilistic projection. It will be shown how the framework can be used to calculate the probability that a given level above the current mean sea level could be flooded sometime within a future time period. A number of examples of different applications are also shown and discussed, but many more could easily be set-up and adapted by future users. A great benefit of the new framework is that is uses only completely free and open data. Mean sea level projections are from the Intergovernmental Panel on Climate Change (IPCC) and extreme sea levels are derived using open data from the Swedish Meteorological and Hydrological Institute.

## Materials and methods

### Mean sea level rise projection

Our mean sea level rise projections for Stockholm are from the IPCC special report: The Ocean and Cryosphere in a Changing Climate (SROCC) (Oppenheimer et al. 2019). These projections exist for three different representative concentration pathways (RCPs) and are complemented with estimates of post glacial rebound from the NKG2016LU model (Vestøl et al. 2019), as described in Hieronymus and Kalén (2020). The SROCC global mean sea level projections for the different RCPs agree well with those produced for the similarly named Shared Socioeconomic Pathways (SSPs) in the IPCC’s Sixth Assessment Report (Fox-Kemper et al. 2021). However, regional projections for Stockholm based on those newer projections were not yet available at the time this article was written.

The RCPs are labelled according to their radiative forcings in the year 2100, which are 2.6, 4.5 and 8.5 ${\text{Wm}^{-2}}$. In RCP2.6, emissions peak around 2020, while in RCP8.5 they continue to grow throughout the century (van Vuuren et al. 2011). The *likely* range of the temperature increase for 2081–2100 relative to 1986–2005 is 0.3–1.7 $^\circ {\text{C}}$ under RCP2.6, 1.1–2.6 $^\circ {\text{C}}$ under RCP4.5 and 2.6–4.8 $^\circ {\text{C}}$ under RCP8.5 (IPCC 2013). The expected sea level change in Stockholm between the years 2021 and 2100 is − 0.16 m under RCP2.6, − 0.03 m under RCP4.5 and 0.24 m under RCP8.5.

**Fig. 1:**
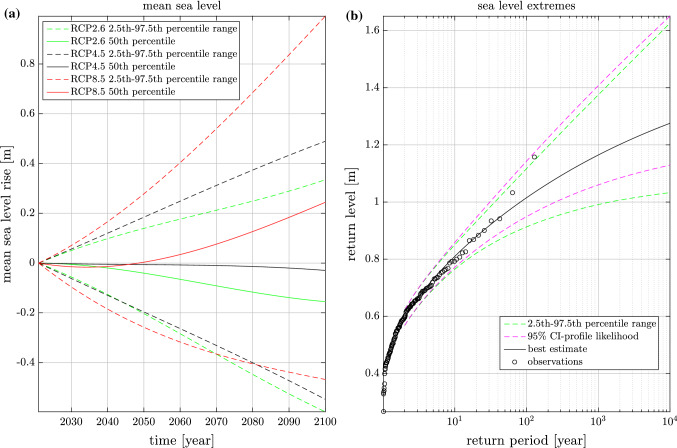
Mean sea level projections and extreme sea level estimates used by the simulator. Mean sea level as a function of time in the different RCPs in (**a**), and return levels for sea level extremes (**b**). The profile likelihood confidence interval is computed directly from the tide gauge data (i.e. the data plotted with black rings in panel **b**). The percentile ranges in **a** are directly computed from the mean sea level rise used in the simulator. The percentile ranges in **b** are computed directly by fitting return level curves to a large amount of random 2021-2100 yearly maximum sea level samples produced by the simulator

The uncertainty in our mean sea level projections is represented by fitting a skew normal distribution to the *likely* range (defined as the range between the 17th and 83rd percentile) of the SROCC regional projection for 2100 interpolated to the Stockholm tide gauge location. In practise we fit the 17th, 50th and 83rd percentile of skew normal distribution to the SROCC distributions values at the 17th minus the 50th percentile, zero and the 83rd minus the 50th percentile. Thus giving us a zero centered uncertainty distribution dependent on the skew normal distributions three parameters: shape ($\xi$), scale ($\sigma$) and location ($\mu$). Each mean sea level projection then gets an uncertain part which is randomly drawn from this distribution and a constant part which is the central estimate projection by Hieronymus and Kalén (2020). The uncertainty in the regional projections is considerably larger than in the more commonly seen globally averaged projections. This is a consequence of many processes averaging out on the global scale (e.g. heat and mass redistribution owing to changes in the ocean circulation), but not in regional projections. The projections used here range from 2021 to 2100 and the uncertainty is set to increase linearly from zero in 2021 to its full value in 2100. The mean sea level projections for the different RCPs and their 2.5th-97.5th percentile ranges are shown in Fig. 1a.

### Extreme sea levels

Extreme sea level distributions are derived by fitting a generalized extreme value distribution (GEV) to the annual maximum time series from the Stockholm tide gauge, using the same data and methodology as in Hieronymus and Kalén (2020). The GEV distribution depends on three parameters: shape ($\xi$), scale ($\sigma$) and location ($\mu$). The tide gauge time series is linearly de-trended to remove the joint impacts of mean sea level rise and post glacial rebound. Years are defined as starting in July and ending in June. Baltic sea level extremes are affected by strong low frequency sea level variability in the basin (Hünicke et al. 2015; Johansson and Kahma 2016), but extremes nearly never occur during summer months (Männikus et al. 2020). The redefined year is thus tailored to give rise to independent yearly maxima. In total we have 127 such years and the highest sea level extreme during this period is 116 cm above the mean.

Extreme sea levels projections are done by randomly drawing one yearly maximum from the GEV distribution determined from the tide gauge data for each year between 2021 and 2100. This is essentially the same method used in Hieronymus and Hieronymus (2021) in their first bootstrapping method. A return level curve for sea level extremes, its 95% confidence interval, and the observations they both are based on are shown in Fig. 1b. The figure also shows the 2.5th-97.5th percentile range of return level curves estimated from samples of yearly sea level maxima for the period 2021-2100 produced by the simulator, as an alternative uncertainty quantification.

### The simulator

**Fig. 2:**
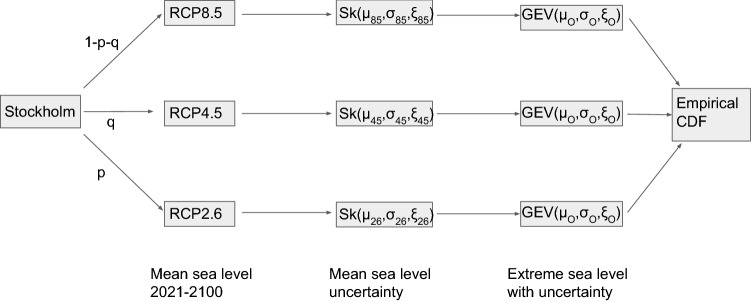
A schematic of the flood risk simulator. The numbers *p* and *q* give the probabilities of the scenarios RCP2.6 and RCP4.5 coming to pass, respectively. The probability of the scenario RCP8.5 is set to be $1-p-q$. The $\xi$, $\sigma$ and $\mu$ parameters of the skew normal mean sea level uncertainty are RCP dependent and fitted to SROCC projections of Oppenheimer et al. (2019), while those for the GEV are determined from the tide gauge data and are assumed to be independent of the RCP scenario

The purpose of the simulator is to incorporate mean sea level rise and sea level extremes into one joint probabilistic framework. A schematic of the framework is shown in Fig. 2. To run the simulator one must first prescribe probabilities for the different RCPs (i.e. *p* and *q*). Current practise in many Swedish municipalities including Stockholm is to use the unlikely, and very cautious, choice of $p=0,q=0$, which gives RCP8.5 the probability of one. In reality *p* and *q* are essentially unknowns, and the whole representation of future greenhouse gas concentrations is somewhat hampered by the fact that we only have projections for three RCPs. Nevertheless, using all three certainly allows a more realistic representation of possible future emissions than only keeping the highest emission scenario. Moreover, the framework can be used to assess how the assumed scenario probabilities affect the risk of flooding, which is another major improvement over current practise where scenario uncertainty is often simply ignored.

The simulator produces an empirical cumulative distribution function (CDF) that gives the probability that a certain level, *x*, above the current mean sea level will be flooded at least once between the years 2021 and 2100. We call this function *F*(*p*, *q*, *x*). The probabilities are found by running the simulator very many times (we call the amount of ensemble members, or 2021-2100 periods, *n*). Our main results are derived using $n=10^9$, meaning that the period 2021–2100 is simulated $10^9$ times for each different [*p*,*q*] combination. Each of these *n* ensemble members has its own mean sea level projection for the period with a random component drawn from the skewnormal distribution and its own unique set of yearly maximum sea levels drawn from the GEV distribution. The GEV based yearly maximum sea levels have the local mean sea level as reference and are thus added to the yearly mean sea level projection to find the yearly maximum sea level relative to the 2020 mean sea level. The CDF value for any given level above the mean is then computed by dividing the amount of ensemble members that reached that particular level by the total amount of ensemble members.

Using the same technique and ensemble; a CDF called *G*(*p*, *q*, *t*), where *t* is time in years, is also computed. This CDF gives the probability for when during the 2021-2100 period the highest sea level occurs. Here CDF values are found by dividing the amount of ensemble members who had their highest sea level reached before or during the year *t* by the total amount of ensemble members. The simulator set-up is the same throughout the article except for some smaller modification such as adding an adaptation option and embedding the framework into a simple decision problem. These smaller modifications are described separately in the results section.

## Results

### Simulator results and their uncertainties

**Fig. 3:**
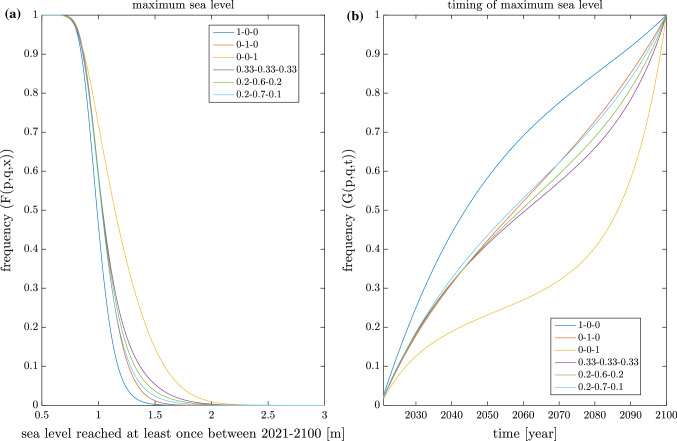
Maximum sea level and timing of maximum sea level CDFs. The number triplets in the legends give the values of *p*, *q* and $1-p-q$. The CDFs are calculated from an ensemble of $10^9$ 2021–2100 periods

Figure 3 shows the CDF of sea levels reached at least once during the period 2021–2100 for some different [*p*, *q*] combinations, as well as the CDF of the year when the highest sea level is expected to occur. The legend shows the $p/q/(1-p-q)$ numbers used in the different experiments. The maximum sea levels reached in the $10^9$ member ensembles together with the probability that the 2.5 m level (the current minimum height above sea level where new constructions are allowed) will be flooded are given in Table 1. A nice feature of this framework is that the somewhat arbitrarily chosen 2.5 m level can now be given a probabilistic meaning. It is, for example, abundantly clear that the 2.5 m level is highly unlikely to be reached during the projection period even under RCP8.5 ($p=0,q=0$), while its almost impossibly unlikely under RCP2.6. It is also evident from the table that the risk of seeing a 2.5 m sea level reached this century is almost uniquely determined by the probability of having emissions following RCP8.5. This follows from the nearly perfect scaling of $1-p-q$ with the probability of exceeding the 2.5 m level (see Table 1).

The timing of the extremes also reveal some interesting scenario dependent features. Again it is RCP8.5 (RCP2.6) that sticks out with a very high (low) probability of seeing the highest sea levels late in the century. This is, of course, a function of the difference in the expected mean sea level change in the two scenarios. Under RCP8.5 the expected sea level rise exceeds the post glacial rebound so the relative sea level is expected to rise, while the opposite is true under RCP2.6.

**Fig. 4:**
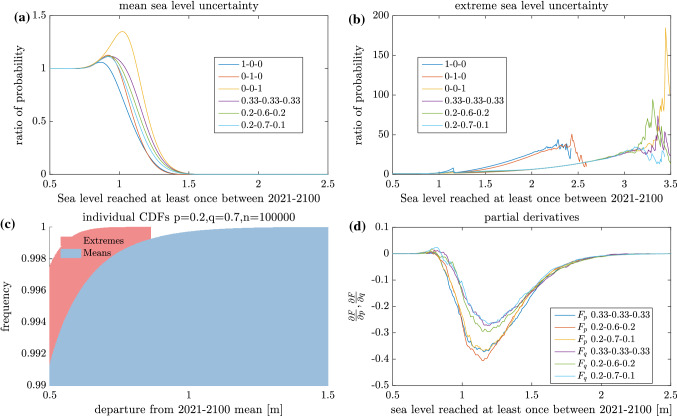
Mean and extreme sea level uncertainty quantifications. **a** ratio of CDF without mean sea level uncertainty to reference case, **b** ratio of CDF without extreme sea level uncertainty to reference case, **c** CDFs of departure from 2021–2100 of mean and extreme sea level and **d** partial derivatives of *F* with respect to *p* and *q*. $n=1\times 10^9$ is used in **a**, **b** and **d**. The designation $F_p$ stands for $\partial F/\partial p$ and similarly for $F_q$

To better understand how the mean sea level and extreme sea level uncertainty respectively contribute to the ensemble spread we have conducted some additional experiments. Figure 4a, shows a CDF computed without mean sea level uncertainty (i.e. each ensemble member gets the central estimate mean sea level projection from Hieronymus and Kalén (2020)) divided by that from the reference case shown in Fig. 3. This CDF ratio becomes extremely small for high sea levels, suggesting that the most extreme sea levels are practically impossible to occur without a very extreme mean sea level projection, far exceeding even the expected mean sea level rise under RCP8.5.

The extreme sea level uncertainty is similarly quantified in panel (b). Here a CDF is computed so that the random GEV sea level extremes are replaced each year with the highest recorded sea level above the mean (1.16 m from the Stockholm tide gauge). This CDF is then divided by the CDF from the reference case as was done in the mean sea level case in panel (a). In this experiment the probability of seeing extremely high sea levels in Stockholm becomes greatly amplified. This happens even though the GEV distributed extremes used in the reference case can be significantly higher than 1.16 m above the mean. However, such extremes are infrequent. With the current GEV fit the yearly probability of hitting the highest recorded sea level from the tide gauge is about 1/871. In light of this one might argue that a better way of removing extreme sea level uncertainty would be to take the mean of the yearly maximum values. However, the current approach is tailored to be relevant for coastal planners. Both the maximum recorded sea level and events with a yearly probabilities much lower than 1/871 are currently used to determine building free levels in different European countries, while the average yearly maximum sea level is not (Fredriksson et al. 2016; Boverket 2020).

The reason why mean sea level rise rather than infrequent very high sea level extremes is responsible for the highest sea levels modelled by the simulator is illustrated in panel (c). Here the right tail of the CDFs of the departure from the 2021-2100 mean of the mean sea level and the sea level extremes are shown. That is, the CDFs depict the variation of the yearly maximum sea level and the yearly mean sea level around their respective ensemble means, and we are looking at the probability range between 0.99 and 1. It is clear that for Stockholm, the right tail of the distributions is much longer for mean sea levels than for sea level extremes, making mean sea level rise much more likely to cause high sea levels. Note that this is true even in the case studied here where $p=0.2$ and $q=0.7$. Meaning that the probability, 1/10, of getting RCP8.5 is much less than those for getting RCP4.5 or RCP2.6 of respectively 7/10 and 2/10.

The uncertainty owing to the choice of RCP scenario probabilities can be quantified approximately using Taylor expansions. Here we make numerical differentiations of *F*(*p*, *q*, *x*) with respect to *p* and *q*. These derivatives can then be used to approximate *F*(*p*, *q*, *x*) for small changes in *p* and *q* using such an expansion. To first order we get,

1 $$\begin{aligned}&F(p_0+\varDelta p,q_0+\varDelta q,x)\approx F(p_0,q_0,x)+\frac{\partial F}{\partial p}(p_0,q_0,x)\varDelta p\nonumber \\&\quad +\frac{\partial F}{\partial q}(p_0,q_0,x)\varDelta q, \end{aligned}$$

where $p_0$ and $q_0$ are any set of currently used estimates of *p* and *q*. Figure 4d illustrates $\partial F/\partial p$ and $\partial F/\partial q$ for some different combinations of *p* and *q*. In general it is clear that $\partial F/\partial p<\partial F/\partial q$. This is natural since increasing *p* means increasing the likelihood of RCP2.6 on the expense of the likelihood of RCP8.5, while in the case of *q* it is the more similar RCP4.5 and RCP8.5 scenarios that have their probabilities perturbed in equal amounts. It is clear from the figure that both partial derivatives have well defined minima between sea levels reached of 1 and 1.5 m. It is thus in this region where *F*, in absolute terms, is most strongly affected by changes in *p* and *q*. It is also clear that while *F* is sensitive to variations in *p* and *q* (see Fig. 3), ${\partial} F/ {\partial} p \; {\text{and}} \; {\partial} F/ {\partial} q$ are both quite similar for the different [*p*, *q*] combinations probed here. The effect of changing *p* and *q* by some given amount on *F* is thus relatively independent of the $p_0$ and $q_0$ values used for a large range of *x* values.

### Adaptation and decision problems

In this subsection we present some simple applications of the simulator. The first application is an adaptation problem. Here we imagine that we have a settlement or intend to build one at some given height above today’s mean sea level. We also imagine that we could protect this settlement from flooding, for example, by building a sea wall. However, protection is expensive and unnecessary with today’s mean sea level. We therefore decide to model at what mean sea level increase we would have to build the wall to keep our flooding risk at a suitably low level. For this experiment we use $p=0,q=0$ as RCP8.5 is the only one of our scenarios where such adaptation measures could reasonably become practicable during this century. The set-up of the simulator is the same as that used in Fig. 3, but $n=10^8$ is used here to save some computational time. However, an extra condition on each 2021–2100 period is added in this application. That is, that when the mean sea level has risen by $\varDelta H$ m the local decision-makers decide to protect the settlement. Getting such protection agreed upon and built, however, could not reasonably be expected to occur within the year even if the decision-makers lives depended upon it. The protection is thus assumed to be active first 10 years after the mean sea level has exceeded $\varDelta H$. When the protection is up all subsequent yearly maxima are set to zero and the CDF is computed as in the reference case.

**Fig. 5:**
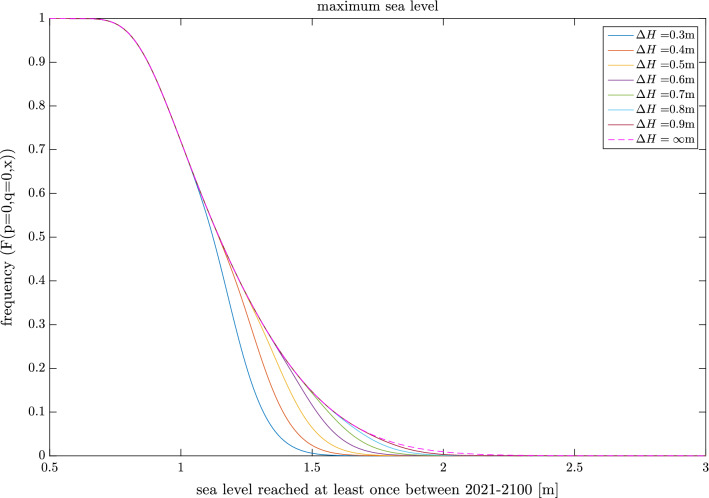
Same as Fig. 3a, but with adaptation option for some different levels of $\varDelta H$. $\varDelta H=\infty$ shows the no adaptation option $n=1\times 10^8$ is used

Figure 5 shows the CDF from the adaptation application for some values of $\varDelta H$. The $\varDelta H=\infty$ case shows the no adaptation CDF. Some results are also summarized in Table 2. It is abundantly clear from the experiment that such protection measures could very effectively lower the probabilities of seeing high sea levels. Moreover, it is clear that the current 2.5 m building free level could be protected efficiently even with a high $\varDelta H$. This owes in part to the very low probability that such a level could be reached in this century even without protection. The high efficiency of these measures is, however, also dependent on the fact that for Stockholm very high sea levels are almost exclusively owing to very high mean sea level rise, not extremely rare high extremes (see Fig. 4a, b and c). Conditioning protection on mean sea level rise would therefore be very efficient in Stockholm. In other locations the situation can be different.

**Fig. 6:**
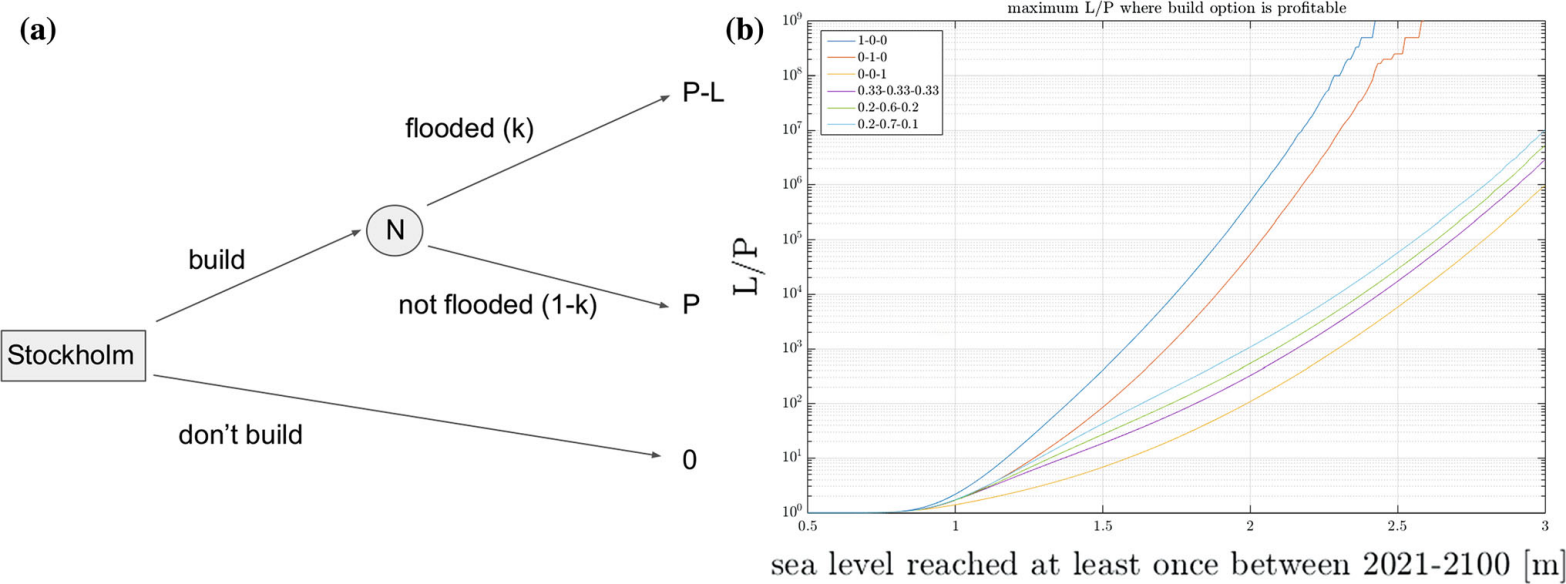
Schematic of the decision problem (**a**) and maximum L/P ratios where build option is profitable (**b**). The node name *N* in **a** stands for nature and and is represented by the simulator who calculates the probability *k*. The probability of flooding, *k*, used in **b** panel is thus consistent with that in Fig. 3a)

The last application showcased here is a decision problem. Here we imagine that the 2.5 m rule does not exist and that we want to find a suitably high level above the mean sea level, where the benefits of developing land outweighs the risk of it being flooded. A schematic of this problem is shown in Fig. 6a. The objective is thus for Stockholm to decide on whether to develop some land situated *x* m above the 2020 mean sea level. Developing the land would yield a profit *P*, the same profit would, of course, not be realized if the land was not developed. In case the land is developed there is some risk, *k*, that the land is flooded during the 2021–2100 period, which leads to a loss *L*. There is also a chance $1-k$ that the land is not flooded in which case the profit *P* is realized. For the development option to be attractive the expected profit from developing the land must be greater than zero. Thus, in this simple example we get the condition,

2 $$\begin{aligned} 0<(1-k)P+k(P-L) \end{aligned}$$

or equivalently

3 $$\begin{aligned} \frac{L}{P}<\frac{1}{k}. \end{aligned}$$

Figure. 6b shows the maximum *L*/*P* ratio where the building option is preferable as a function of height above the sea for some different [*p*, *q*] combinations. For the current 2.5 m level, it is clear that, developing land is profitable in the different scenarios used here as long as *L* does not exceed *P* by more than four to nine orders of magnitude.

## Discussion

The simulator results depend on a number of different assumptions that are hard or even impossible to test; such as the values of *p* and *q* and also the assumed distributions for Stockholm’s future mean sea levels and sea level extremes. Mean sea level projections rely to a large degree on subjective expert judgements and especially the uncertainty estimates in different high emission projections can be very different (Jevrejeva et al. 2018; Horton et al. 2018; Le Bars 2018; Hieronymus 2020). The mean or median projections for mean sea level are typically in better agreement than the uncertainty quantifications since those more often derive directly from model results. Generally speaking different projections for low and medium emission scenarios typically agree much better than those for high emission scenarios and the agreement is also much higher in shorter projections (Fox-Kemper et al. 2021). Nevertheless, exchanging the current SROCC based mean sea level projection for some other projection could have large effects on the computed probabilities and simulators should thus be updated with some regularity. For the present it is worth noting that the new main projections from IPCC’s sixth assessment report (Fox-Kemper et al. 2021) agree well, at least, in their global averages with those from SROCC. However, the sixth assessment report also includes projections that take their ice sheet contribution to sea level rise from an expert judgements study and a single ice sheet model for Antarctica which incorporates parametrized marine ice cliff instability. Those projections give considerably higher mean sea level rise under the highest emission scenario (Bamber et al. 2019; DeConto et al. 2021; Fox-Kemper et al. 2021). Including such scenarios in the simulator would lead to an even greater dominance of mean sea level uncertainty over extreme sea level uncertainty, and of course to higher probabilities for seeing high see levels.

Sea level extremes also come with considerable uncertainties, both methodological and owing to relatively short high resolution observational records (Suursaar and Sooäär 2007; Dangendorf et al. 2016; Wahl et al. 2017; Hieronymus and Hieronymus 2021). For Stockholm, however, the high resolution observational record is, in fact, one of the longest in the world (Ekman 1999), lending further support for the conclusion that for Stockholm the extreme sea level uncertainty is much less important than the mean sea level uncertainty. This conclusion is, however, somewhat at odds with the recommendations of the Swedish National Board of Housing, Building and Planning. In their “Starting points for flood risk assessment” (translation of Swedish original title: Utgångspunkter för bedömning av översvämningsrisk) they suggest that developments of new important infrastructure and settlements should be done with a 10 000 year return level in mind. At the same time they give no exact recommendation for which RCP based mean sea level projection or percentile thereof to use for planning, apart from suggesting that RCP8.5 can often be useful (Boverket 2020). Our simulator results, however, clearly suggest that, at least, in Stockholm high sea levels are much more likely to occur as a consequence of high mean sea level rise than as a consequence of a very extreme storm surge, even when RCP8.5 is given the probability of 1/10. Thus, adding a very extreme 1/10000 year event to a central estimate RCP8.5 projection does not appear as a particularly great recipe. These conclusions are, of course, specific to Stockholm. However, it does seem highly unlikely, given that both mean sea level rise and sea level extremes are quite consistent along large parts of the Swedish coasts, that adding a 10000 year return level to an RCP projection would generally give rise to good planning levels. Instead, our results suggest that high percentile mean sea level rise should be given more thought in planning than high percentile extremes and that site specific recipes are likely needed.

Our simulator results are very sensitive to the values used for *p* and *q* and in particular to the value of $1-p-q$ which give the probability for RCP8.5 to occur. This is readily seen from Fig. 4d. Most experts believe RCP8.5 to be a very unlikely future, even as a baseline scenario where no new environmental policies are put in place (van Vuuren et al. 2011; Hausfather and Peters 2020). With this in mind it could seem a strange choice that the lowest non-zero value of $1-p-q$ discussed here is 1/10. One reason for the scenario choices made here is the lack of sea level projections for RCPs between 4.5 and 8.5, and $1-p-q$ is thus set somewhat high to compensate. Future simulators would benefit greatly both from having more scenarios and better constrained scenario probabilities. In the mean time, however, the simulators usage of scenario probabilities and our ability to use simulators to analyse the effects of varying these probabilities is a major improvement over current practice where most often just a single high emission scenario is used (Department of civil engineering county administrative board of Stockholm 2015; Boverket 2020). Another smaller caveat for some application might be that only yearly maximum sea levels are modelled. Under some RCP8.5 realisations, for example, it is quite likely that even a second or third highest sea level in one year late in the century could be one of the highest recorded throughout the whole century. However, for most applications only locations where the probability of coastal flooding is extremely low would be considered for any development plans, so this unlikely to be a major problem.

The adaptation problem shown in Fig. 5 and the decision problem shown in Fig. 6 are both simple and direct applications using the simulator. The adaptation problem clearly shows that the probabilities of flooding can be very effectively reduced with protective measures that are installed once the mean sea level has risen by some given amount. That is, assuming that the protection works properly and can be built in ten years. The decision problem is highly simplified, being a single-person decision problem (Tadelis 2013), while in reality there are many stakeholders making profits and losses when areas are developed or flooded. It is also simplified in that the options available to the player is only build or do not build. In reality a merger of the decision and adaptation problems would give rise to the perhaps more attractive build and protect option. Such extensions can easily be made using the tools presented and discussed here. The biggest challenge with the decision problem, at least to a natural scientist, however, is to produce reasonable estimates of *P* and *L*. This issue has been avoided here where only bounds on their ratio are computed, but this would be useful extension in future work. To make reasonable *P* and *L* estimates one has to tackle tricky issues on, for example, the valuation of settlements and one must also decide to what extent future losses should be discounted (Nordhaus 2007).

One further caveat worth mentioning is that for some developments the time frame 2021–2100 may be too restrictive, especially, if one believes high emission scenarios to be likely. Since then sea level maxima are much more likely to occur toward the end of the century, see Fig. 3b, and flooding risk are thus far from stationary. Even without mean sea level rise we might expect some non-stationarity in the extremes (Kudryavtseva et al. 2018), but this issue gets greatly exacerbated with mean sea level rise occurring over long periods. The simulator’s projection period can be extended in the future when more long sea level projections become available. However, if multi-centennial sea level projections are used, scenario probabilities (i.e. *p* and *q*) would be extremely hard to estimate. More different scenarios such as overshoots and high emission scenarios that turn into rapid emission reduction scenarios would also be very useful to better represent the great uncertainty in future emission pathways.

## Conclusions

A yearly maximum sea level simulator for Stockholm has been presented. The simulator is a natural framework for incorporating emission scenario-, mean sea level- and extreme sea level uncertainty into one unified probabilistic framework. The framework is, however, avant-garde given that even just the use of probabilistic mean sea level projections is uncommon in Europe today. In a 2021 survey of 32 European countries, McEvoy et al. (2021) found that probabilistic mean sea level information was only used in dedicated sea level planning in a single country and in non-dedicated planning in a further three. Current practice in Swedish municipalities and also in many places outside of Sweden is that new buildings and infrastructure must be placed at a distance deemed safe some *x* m above the current mean sea level. The level *x* used is often a rather arbitrary construct typically derived by adding some high return level to some projected future mean sea level (Arns et al. 2017; Department of civil engineering county administrative board of Stockholm 2015; Boverket 2020). The latter is most often the mean sea level for year 2100 under RCP8.5, both in a Swedish and a European context (McEvoy et al. 2021). The risk that the level *x* could become flooded some time between now and the year 2100 is typically not assessed. Moreover, the uncertainties of the estimates adding to the sum *x* are typically not treated in a consistent way, and sometimes they are not even considered at all (McEvoy et al. 2021). The here proposed framework resolves, or at least alleviates both these problems and thus constitutes a significant improvement over current practise.

Another major benefit of the framework is that uncertainties can be manipulated and analysed in a straightforward manner. For Stockholm this leads to the conclusion that mean sea level uncertainty contributes much more to the overall uncertainty than extreme sea level uncertainty. This result is, of course, site specific owing both to Stockholm having relatively modest sea level extremes (Hieronymus et al. 2018) and low extreme uncertainty because of its long observation time series (Ekman 1999). However, Stockholm also experiences a considerable land rise that counteracts the mean sea level rise, making the mean sea level rise there slower than in many other locations. Thus, a dominance of mean sea level uncertainty over extreme sea level uncertainty might be expected along much of the Swedish coast where the post glacial rebound is slower than in Stockholm.

Lastly, the framework is also versatile and easily adapted as was showcased in the adaptation and decision problem applications. While both those applications are highly simplified they illustrate well how the natural science ingredients of the problem, that is, mean sea level projections and extreme sea level estimates can be used in a natural way to tackle decisions problems of relevance for coastal communities around the world today.

**Table 1:** Maximum sea level reached and probability of exceeding a sea level of 2.5 m above the 2020 mean in the experiments of Fig. 1. The number triplets in the first row give the value of the probabilities $p-q-(1-p-q)$. Ensemble size is $n=1\times 10^{9}$

	1–0–0	0–1–0	0–0–1	0.33–0.33–0.33	0.2–0.6–0.2	0.2–0.7–0.1
Maximum SSH	2.43	2.60	3.50	3.55	3.66	3.44
Probability 2.5 m	0	$4.0\times 10^{-9}$	$1.8\times 10^{-4}$	$5.9\times 10^{-5}$	$3.5\times 10^{-5}$	$1.8\times 10^{-5}$

**Table 2:** Maximum sea level reached and probability of exceeding a sea level of 2.5 m above the 2020 mean in the adaptation experiments of Fig. 5. The parameters used for the experiment is $p=q=0$ and $n=1\times 10^{8}$

	$\Delta H=0.3$	$\Delta H=0.4$	$\Delta H=0.5$	$\Delta H=0.6$	$\Delta H=0.7$	$\Delta H=0.8$	$\Delta H=0.9$
Maximum SSH	1.88	2.02	2.11	2.19	2.30	2.39	2.52
Probability 2.5 m	0	0	0	0	0	0	$0.2\times 10^{-7}$

## References

[CR1] Arns A, Dangendorf S, Talke JJS, Bender J, Pattiaratchi C (2017). Sea-level rise induced amplification of coastal protection design heights. Scientific Reports.

[CR2] Bamber JL, Oppenheimer M, Kopp RE, Aspinall WP, Cooke RM (2019). Ice sheet contributions to future sea-level rise from structured expert judgment. Proceedings of the National Academy of Sciences.

[CR3] Boverket. 2020. Utgångspunkter för bedömning av översvåmningsrisk. https://www.boverket.se/sv/PBL-kunskapsbanken/planering/detaljplan/lansstyrelsens-tillsyn/tillsynsvagledning_naturolyckor/tillsynsvagledning-oversvamning/stod-till-lansstyrelsen-vid-riskbedomning/utgangspunkter/.

[CR4] Dangendorf S, Arns A, Pinto JG, Ludwig P, Jensen J (2016). The exceptional influence of storm ‘Xaver’ on design water levels in the German Bight. Environmental Research Letters.

[CR5] DeConto RM, Pollard D, Alley RB, Velicogna I, Gasson E, Gomez N, Sadai S, Condron A (2021). The Paris Climate Agreement and future sea-level rise from Antarctica. Nature.

[CR6] Department of civil engineering county administrative board of Stockholm. 2015. Rekommendationer för lägsta grundläggningsnivålängs Östersjökusten i Stockholms län – med hänsyn till risken för översvämning. Tech. rep., County administrative board of Stockholm, Länsstyrelsen i Stockholms län Avdelningen för samhällsbyggnad.

[CR7] Ekman M (1999). Climate changes detected through the world’s longest sea level series. Glob and Plan Change.

[CR8] Fox-Kemper, B., H.T. Hewitt, C. Xiao, G. Aðalgeirsdóttir, S.S. Drijfhout, T.L. Edwards, N.R. Golledge, M. Hemer, et al. 2021. In *Ocean, cryosphere and sea level change*. in press.

[CR9] Fredriksson C, Tajvidi N, Hanson H, Larson M (2016). Statistical analysis of extreme sea water levels at the Falsterbo Peninsula, South Sweden. Journal of Water Management and Research.

[CR10] Hausfather Z, Peters GP (2020). Emissions—The ‘business as usual’ story is misleading. Nature.

[CR11] Hieronymus M (2020). Nonlinear interactions and some other aspects of probabilistic sea level projections. Water.

[CR14] Hieronymus M, Dieterich C, Andersson H, Hordoir R (2018). The effects of mean sea level rise and strengthened winds on extreme sea levels in the Baltic Sea. Theoretical and Applied Mechanics Letters.

[CR13] Hieronymus M, Kalén O (2020). Sea-level rise projections for Sweden based on the new IPCC special report: The ocean and cryosphere in a changing climate. Ambio.

[CR12] Hieronymus M, Hieronymus F (2021). Southern Baltic sea level extremes: Tide gauge data, historic storms and confidence intervals. Boreal Environmental Research.

[CR15] Hinkel J, Aerts J, Brown S, Jiménez JA, Lincke D, Nicholls RJ, Scussolini P, Sanchez-Arcilla A, Vafeidis A, Addo KA (2018). The ability of societies to adapt to twenty-first-century sea-level rise. Nature Climate Change.

[CR16] Horton BP, Kopp RE, Garner AJ, Hay CC, Khan NS, Roy K, Shaw TA (2018). Mapping sea-level change in time, space, and probability. Annual Review of Environment and Resources.

[CR17] Hünicke B, Zorita E, Soomere T, Madsen KS, Johansson M, Suursaar Ü (2015). Recent change-sea level and wind waves.

[CR18] IPCC. 2013. Summary for Policymakers, Cambridge University Press, Cambridge, United Kingdom and New York, NY, USA, book section SPM, 1–30. 10.1017/CBO9781107415324.004, www.climatechange2013.org.

[CR19] Jevrejeva S, Frederikse T, Kopp RE, Jackson GLCLP, van de Wal RSW (2018). Probabilistic sea level projections at the coast by 2100. Surveys in Geophysics.

[CR20] Johansson M, Kahma KK (2016). On the statistical relationship between the geostrophic wind and sea level variations in the Baltic sea. Boreal Environment Research.

[CR21] Kudryavtseva N, Pindsoo K, Soomere T (2018). Non-stationary modeling of trends in extreme water level changes along the Baltic Sea coast. Journal of Coastal Research.

[CR22] Le Bars D (2018). Uncertainty in sea level rise projections due to the dependence between contributors. Earth’s Future.

[CR23] Männikus R, Soomere T, Viŝka M (2020). Variations in the mean, seasonal and extreme water level on the Latvian coast, the eastern Baltic Sea, during 1961–2018. Estuarine, Coastal and Shelf Science.

[CR24] McEvoy S, Haasnoot M, Biesbroek R (2021). How are European countries planning for sea level rise?. Ocean & Coastal Management.

[CR25] Mitrovica JX, Hay CC, Kopp RE, Harig C, Latychev K (2018). Quantifying the sensitivity of sea level change in coastal localities to the geometry of polar ice mass flux. The Journal of Climate.

[CR26] Nordhaus WD (2007). A review of the Stern review on the economics of climate change. Journal of Economic Literature.

[CR27] Oppenheimer, M., B. Glavovic, J. Hinkel, R. van de Wal, A.K. Magnan, A. Abd-Elgawad, R. Cai, M. Cifuentes-Jara, et al. 2019. In *Sea level rise and implications for low lying islands, coasts and communities*. in press.

[CR28] Schöld, S., I. Gudmundsson, L. Lind, M. Hieronymus, A. Jönsson, and E. Breviere. 2020. *Hur kan sverige rusta för stigande hav??: Sammanfattning och slutsatser från workshop on sea level rise*. Swedish Meterological and Hydrological Institute: IPCC SROCC science and planning for climate adaptation. Tech. rep.

[CR29] Suursaar Ü, Sooäär J (2007). Decadal variations in mean and extreme sea level values along the Estonian coast of the Baltic Sea. Tellus A: Dynamic Meteorology and Oceanography.

[CR30] Swedish Meterological and Hydrological Institute. 2021. Framtida medelvattenstånd. https://www.smhi.se/klimat/stigande-havsnivaer/framtida-medelvattenstand-1.165493.

[CR31] Tadelis S (2013). Game theory an introduction.

[CR32] Vestøl O, Ågren J, Steffen H, Kierulf H, Tarasov L (2019). NKG2016LU: A new land uplift model for Fennoscandia and the Baltic region. Journal of Geodesy.

[CR33] van Vuuren DP, Edmonds J, Kainuma M, Riahi K, Thomson A, Hibbard K, Hurtt GC, Kram T (2011). The representative concentration pathways: An overview. Climatic Change.

[CR34] Wahl T, Haigh ID, Nicholls RJ, Arns A, Dangendorf S, Hinkel J, Slangen AB (2017). Understanding extreme sea levels for broad-scale coastal impact and adaptation analysis. Nature Communications.

